# Live-cell 3D-SIM of Rift Valley fever virus NSs filaments reveals a polygon web architecture

**DOI:** 10.1073/pnas.2534404123

**Published:** 2026-03-16

**Authors:** James I. Dunlop, Peter A. Thomason, Leo M. Carlin, Benjamin G. Davis, Stephen D. Carter

**Affiliations:** ^a^Medical Research Council-University of Glasgow Centre for Virus Research, School of Infection & Immunity, Glasgow G61 1QH, United Kingdom; ^b^Cancer Research UK Scotland Institute, Glasgow G61 1BD, United Kingdom; ^c^School of Cancer Sciences, University of Glasgow, Glasgow G12 0ZD, United Kingdom; ^d^The Rosalind Franklin Institute, Didcot OX11 0QX, United Kingdom; ^e^Department of Chemistry, Chemistry Research Laboratory, University of Oxford, Oxford OX1 3TA, United Kingdom

**Keywords:** nucleus, super-resolution microscopy, in situ structural biology

## Abstract

A defining feature of Rift Valley fever virus (RVFV) is the incorporation of the NSs protein into large filamentous assemblies inside infected nuclei [R. Swanepoel, N. K. Blackburn, *J. Gen. Virol.* **34**, 557–561 (1977).], as judged from fixed specimens. To gain insight into the 3D structure of NSs filaments within live-cell nuclei, we used genetic-code expansion (GCE) to incorporate trans-cyclooct-2-en-L-lysine into the protein. This enabled site-specific fluorescent labeling with tetrazine dyes for live-cell structured illumination microscopy (SIM). Our superresolved images revealed the complete native architecture of NSs filaments as a micron-scale polygon web of fibers with discrete domain characteristics, overturning previous assumptions of simple linear filaments. Parallel experiments on fixed RVFV-infected cells confirmed that native NSs filaments also display this morphology. Overall, our 3D-SIM analysis reveals distinct structural plasticity within NSs filaments, establishing a quantitative structure–function relationship that support the importance of polygon organization for NSs filament function during RVFV infection.

Rift Valley fever virus (RVFV) causes severe disease across North Africa and the Arabian Peninsula (1) and is a WHO Blueprint priority pathogen. RVFV pathogenesis is driven by the nonstructural protein NSs, which forms nuclear filaments (2) that assemble with FBXO3 E3 ligase to degrade TFIIH and PKR (3, 4), crucial for virulence (5–7).

Thin-section TEM revealed NSs filaments as bundles of loosely packed, parallel 10 nm filaments (8, 9), supported by X-ray crystallography (8) and cryo-EM (4). The cryo-EM structure showed right-handed filaments with three NSs–NSs interface types crucial for assembly.

3D-SIM can be conducted with either live or fixed samples. Fixation can distort important structural features, and permeabilization is required for antibodies to access their intracellular targets. To avoid further disrupting NSs architecture with genetically encoded fluorescent proteins, we instead used genetic-code expansion (GCE) to label NSs with a bright, minimally disruptive uAA-fluorophore. This approach enabled 3D-SIM imaging of intact NSs filaments in live-cells. This allowed us to elucidate the native micron-scale architecture of NSs filaments. Using AI-powered segmentation (10, 11), we uncovered an intricate web of NSs fibers that show discrete architectures such as “sheet,” “chain,” and “twisting” morphology.

## Results

**Fig. 1.**
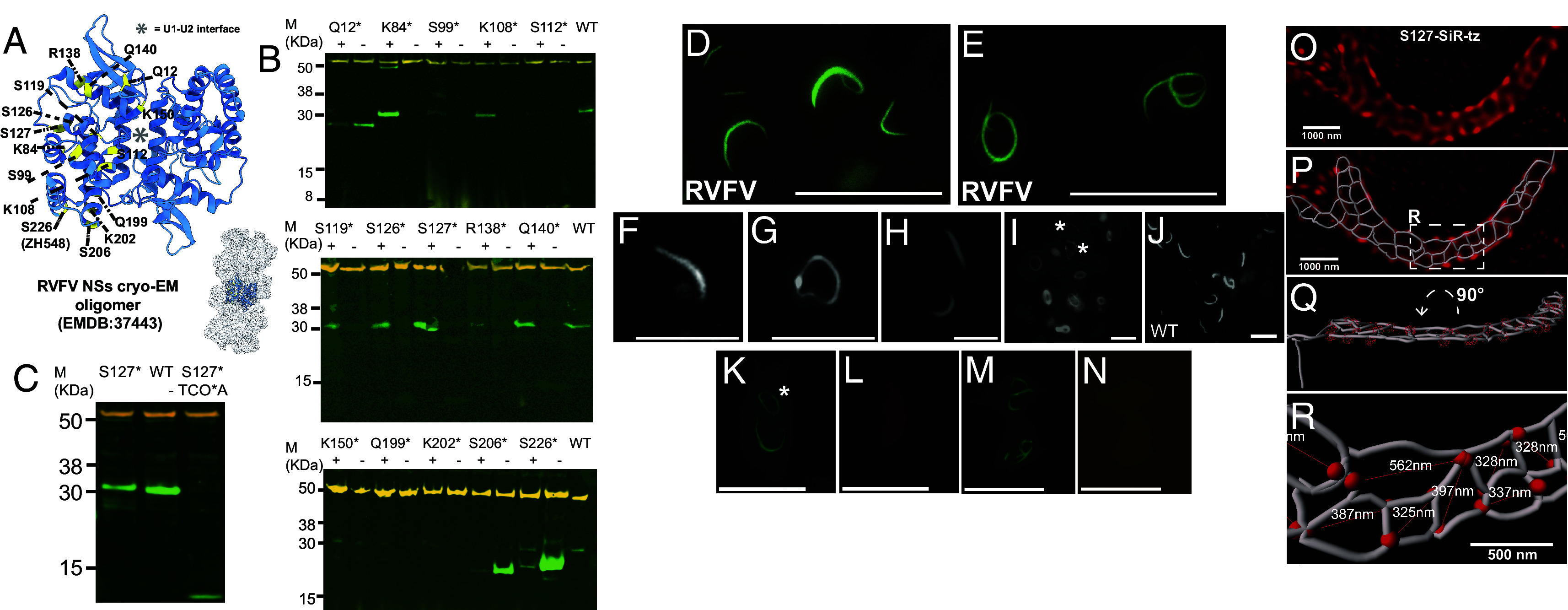
(*A*) RVFV NSs oligomer (4). The NSs–NSs dimer is highlighted as a blue ribbon (PDB:8WCM). Stop-codon residues are highlighted in yellow and numbered. (*B*) Western blot analysis of amber-suppression in HEK-293 T cells for NSs variants (denoted as an asterisk). Tubulin in orange. (*C*) Western blot analysis of amber-suppression in HeLa cells for S127-TCO*A (S127*). (*D*–*E*) Control experiment demonstrating WT RVFV (ZH548) NSs filaments stained with anti-NSs Alexa Fluor® 488 antibody exhibit linear (*D*) and circular morphology (*E*) in VeroE6 cells (58 vs 23 filaments, 81 cells imaged). (Scale bar, 40 µm.) (*F*) Wide-field fluorescence image of Q140*-*CF500 NSs filaments in HEK-293 T cells (33 linear vs 1 circular filaments). (*G* and *H*) Wide-field fluorescence image of S127-TAMRA-tz NSs filaments in HEK-293 T cells (67% linear vs 33% circular, 20 vs 10 filaments, 30 cells imaged). (*I*) Wide-field fluorescence image of S127-CF500 NSs filaments in HEK-293 T cells (53% linear vs 47% circular, 20 vs 18 filaments, 38 cells imaged). Asterisks indicate linear filament morphologies. (*J*) Immunofluorescence of WT NSs filaments in HEK-293 T cells (76 linear vs 18 circular filaments, 94 cells imaged). (*K*–*N*) Control experiment demonstrating TCO*A-dependent labeling. WT NSs lacking TCO*A incorporation were incubated with tetrazine dyes and counterstained with anti-NSs Alexa Fluor® 488 antibody. (*K*) Anti-NSs staining (green). Asterisk indicates circular filament morphology. (*L*) SiR-tz channel (far-red). (*F*–*L*): (Scale bar, 20 µm.) (*M*) Anti-NSs staining (green). (*N*) JF549 channel (yellow). (Scale bar, 25 µm.) Note: Tetrazine channels used longer exposures (1 s vs 5 ms) to visualize diffuse background and confirm absence of specific labeling in the absence of TCO*A across 123 total filaments analyzed (49JF549, 74 SiR-tz). (*O*) Live-cell 3D-SIM analysis of S127-SiR-tz filaments (red) in HeLa cells. (*P*) A segmentation overlaid on the 3D-SIM image shown in *O*. (*Q*) The segmentation in P rotated 90 degrees around the X axis to a more useful perspective. (*R*) Enlarged view of the white dashed inset in P with diameters of the polygons measured in the longest dimension.

We used GCE to incorporate the clickable unnatural amino acid TCO*A (trans-Cyclooct-2-en-L-Lysine) into NSs (12, 13). To guide selection of appropriate amber stop-codon sites, we utilized the RVFV NSs cryo-EM map (EMDB:37443) (Fig. 1*A*). We avoided NSs–NSs interface residues and selected 5 positively charged and 10 polar, uncharged surface-exposed residues that have been shown not to affect NSs function (4). (Fig. 1*A*). To test amber suppression, we expressed each of the 15 NSs variants (strain ZH548) in HEK-293 T cells in the presence of pyrrolysine (Pyl) tRNA and a *M.mazei*-derived tRNACUAPyl synthetase (*SI Appendix*), with and without TCO*A. Cell extracts were analyzed by Western blotting (Fig. 1*B*), indicating that five candidate amber-suppression site variants (K108, S119, S126, S127, Q140) produced abundant, fragment-free protein consistent in size with full-length WT NSs protein in the presence of TCO*A (Fig. 1*B*).

Next, we independently confirmed protein formation by screening for NSs filament assembly using wide-field fluorescence microscopy. To do this, we used bio-orthogonal click chemistry to label NSs variants containing TCO*A at positions K108, S119, S126, S127, and Q140 with CF®500-tetrazine dye. For Q140-TCO*A and S127-TCO*A variants labeled with fluorescent tetrazines, we observed morphological differences in the ratio of linear and circular forms (14). Q140-CF500 showed predominantly linear forms (97% linear, 33 vs 1 filaments) (Fig. 1*F*), while S127 variants across both CF500 and TAMRA dyes displayed both linear and circular morphologies (59% linear vs 41% circular, 40 vs 28 filaments) (Fig. 1 *G*–*I*). This morphological diversity was consistent with WT RVFV (ZH548) NSs filaments in infected VeroE6 cells (72% linear vs 28% circular) (Fig. 1 *D*–*E*) and transiently overexpressed WT NSs (81% linear vs 19% circular) (Fig. 1 *J* and *K*), confirming that our GCE-labeled S127-TCO*A variant represents natural NSs conformational states rather than GCE engineering or labeling artifacts.

**Fig. 2.**
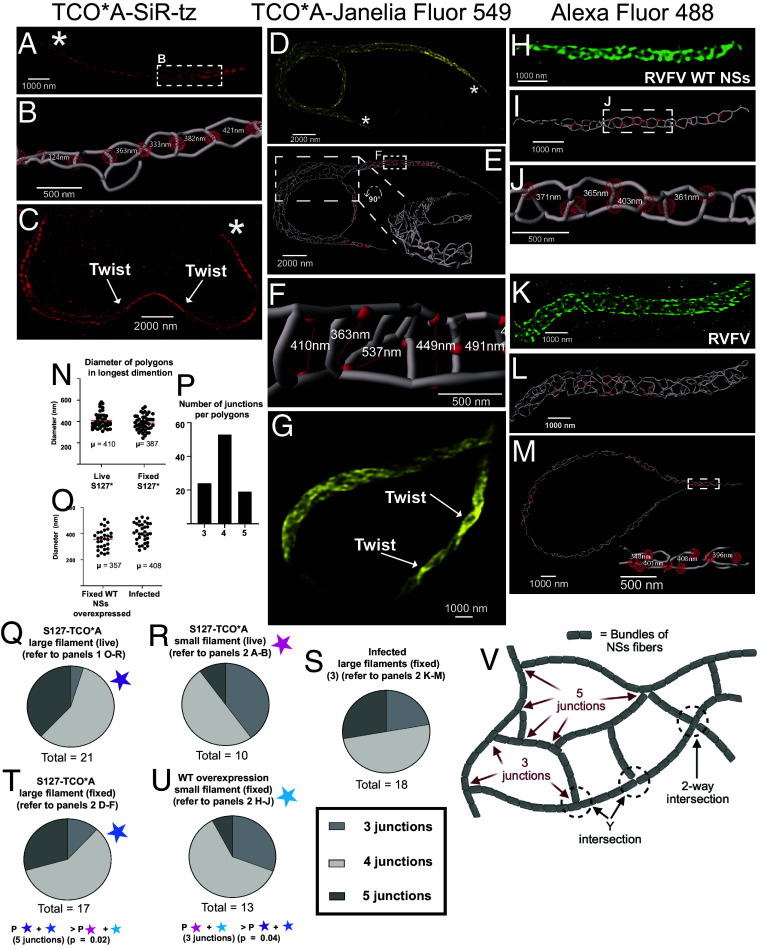
(*A*) Live-cell 3D-SIM of a small S127-SiR-tz NSs filament in HeLa cells (*B*) Segmentation with enlarged view of the white dashed inset in A with selected measurement points represented as red spheres. (*C*) 3D-SIM analysis of a small S127-SiR-tz filament (*D*) 3D-SIM analysis in fixed HeLa cells labeled with Janelia Fluor^®^ 549 (yellow). (*E*) A segmentation of the filament in D. Inset displays the area highlighted within the white dashed box in a more useful perspective and highlights the complex 3D architecture of this filament. (*F*) Enlarged view of the white dashed inset in E with diameters of the polygons measured in the longest dimension. (*G*) 3D-SIM of a large S127-JF549 NSs filament in fixed HeLa cells. (*H*) 3D-SIM analysis in fixed HeLa cells of transiently overexpressed WT filaments. (*I*) A segmentation of *H*. (*J*) Enlarged view of the white dashed inset in I with diameters of the polygons measured in the longest dimension. (*K*) 3D-SIM analysis of RVFV-infected HeLa cells fixed in BSL-3. (*L*) Segmentation with selected measurement points represented as red spheres. (*M*) Segmentation of a second NSs filament from RVFV infected HeLa cells, with selected measurement points represented as red spheres. The inset displays the area highlighted in the white dashed box in a more useful perspective. (*N*) Scatter plot showing diameters of the polygons measured in the longest dimension filaments captured using live-cell (2 filaments) and fixed (1 filament) 3D-SIM. (*O*) Scatter plot showing diameters of the polygons measured in the longest dimension of filaments imaged in RVFV-infected cells (3 filaments) and transiently overexpressed NSs WT fixed (2 filaments). (*P*) Histogram showing the number of polygons made up of 3, 4, and 5 junctions. (*Q*–*U*) Comparison between the number of junctions and filament size. Infected fixed large refers to measurements from three filaments imaged in infected cells, of which two are displayed Fig. 2 *K*–*M*. (*V*) Our proposed model for the NSs polygon web architecture.

Next, we exploited 3D-SIM and stained S127-TCO*A NSs filaments in live-cells with either Janelia Fluor® 549-tetrazine (JF549) or SiR-tz. For this, we used HeLa cells instead of HEK cells, due to their superior imaging properties, after confirming that full-length S127-TCO*A NSs protein was expressed (Fig. 1*C*). To confirm specificity of tetrazine labeling, wild-type NSs filaments expressed without TCO*A were incubated with either JF549 or SiR-tz and counterstained with anti-NSs Alexa Fluor® 488 antibody (Fig. 1 *K*–*N*). While antibody staining confirmed the presence of NSs filaments (green channel), no fluorescence was detected in the yellow (JF549) or far-red (SiR-tz) channels, demonstrating that TCO*A incorporation is required for tetrazine-based labeling (*SI Appendix*). Unexpectedly, both live-cell and fixed 3D-SIM imaging of NSs labeled with SiR-tz and JF549, revealed a large-scale porous web architecture of interconnected filamentous networks. The network contained domains exhibiting intersecting parallel, perpendicular and oblique filaments that produced a series of heterogeneous 3D polygons (Figs. 1*O* and 2*D*) (# of filaments imaged in *SI Appendix*, Table S1). To further characterize network morphology, we applied an AI powered filament tracer tool in Imaris, that delineated an interconnected network of branching fibers and Y-shaped junctions, comprising a polygonal web architecture (Fig. 1 *O*–*R*). This encompassed a variety of morphologies including open lattice structures of flat-sheets (Fig. 1 *O*–*R*), twisting-sheets (Fig. 2*F*), polygon chains (Fig. 2 *A*–*C*), and larger and complex networks (Fig. 2 *D*–*F* and Movie S4), some with twisting morphology (Fig. 2 *C* and *G* and Movies S1 and S2).

We defined “sheets” as containing rows of polygons along the long axis of the filament, sometimes in an alternating series (Fig. 1 *O*–*R* and Movie S3). In contrast, “chains” have a single linear row of organized polygons (Fig. 2 *A*–*C*). In addition, we noticed the “sheet” and “chain” morphology would usually collapse at the extreme ends of the fiber into a compressed coil (Fig. 2 *A*, *C* and *D*, asterisks, Movie S2). Importantly, we observed similar architectures in WT NSs filaments in fixed cells. For example, polygon chains were observed in transiently overexpressed WT filaments (Fig. 2 *H*–*J*), and we also observed large heterogeneous polygon sheet and chain arrangements in RVFV-infected cells that were deactivated under BSL-3 conditions (Fig. 2 *K*–*M*) (# of filaments imaged in *SI Appendix*, Table S1). The presence of these web architectures in WT filaments confirms that these structures are physiologically relevant and not a property of the uAA fluorophore engineering.

Quantitation of NSs polygons demonstrated a mean diameter of 0.410 μm and 0.387 μm for S127-TCO*A-tz live and fixed filaments, respectively, which are consistent with the observed diameters for WT NSs filaments in fixed cells (Fig. 2 *N* and *O* and Movie S3). Interestingly, analysis of polygons in all types of filaments revealed that most polygons contained four Y-shaped junctions, with three and five Y-junction-polygons also present (Fig. 2*P*). We wondered whether the morphological differences between small and large filaments were associated with a change in polygon type. In smaller filaments, whether live or fixed, there were three- to fourfold more 3-Y polygons compared to 5-Y polygons. Conversely, larger filaments contained three- to fourfold more 5-Y polygons than 3-Y polygons (Fig. 2 *Q*–*U*).

## Discussion

Our analysis of native NSs filaments reveals an intricate polygon web architecture with a quantitative structure–function relationship. Polygon complexity correlates with filament size, with smaller filaments containing predominantly 3-Y junction polygons while larger filaments contain approximately threefold more 5-Y junction polygons (Fig. 2 *Q*–*U*), suggesting that polygon formation is important for structural plasticity and organization of NSs filaments.

Furthermore, since it is unlikely that 70° to 90° perpendicular Y-shaped branching occurs within continuous filaments, we suggest a model whereby NSs filaments form polygonal webs via Y-shaped intersections, as seen for actin networks (15) (see our proposed model in Fig. 2*V*). Supporting this model, Li et al. captured possible examples of Y-shaped fiber intersections in their cryo-EM NSs projection images (4).

The biological significance of polygon webs is also supported by Li et al. who demonstrated that complete filament disruption abolishes E3 ligase assembly and blocks degradation of TFIIH and PKR. Since filaments are organized as polygon webs, these functional defects can be understood as consequences of losing this organized architecture.

## Methods

RVFV NSs plasmids containing amber stop codons (pTwist CMV wPRE BG Neo NSs) were cotransfected with pcDNA3.1(+) U6 tRNAPyl_CMV NESPylRS(AF) plasmid encoding pyrrolysine tRNA and *M.mazei*-derived synthetase to incorporate trans-cyclooct-2-en-L-lysine, then labeled with tetrazine dyes for live-cell imaging. 3D-SIM was performed using Zeiss Elyra 7 with 63× objectives and processed via SIM2 algorithms to achieve superresolution visualization of filament architecture. Detailed protocols for genetic code expansion, click chemistry labeling, and image analysis are provided in *SI Appendix*, Methods.

## Supplementary Material

Appendix 01 (PDF)

Movie S1.

Movie S2.

Movie S3.

Movie S4.

## Data Availability

All study data are included in the article and/or supporting information.
