# Supplementary material for: Live-cell 3D-SIM of Rift Valley fever virus NSs filaments reveals a polygon web architecture
Source: Proc Natl Acad Sci U S A. 2026 Mar 16;123(12):e2534404123. doi: 10.1073/pnas.2534404123 (PMC13012112; doi:10.1073/pnas.2534404123)
Supplement: Supplementary file 1 — Appendix 01 (PDF) [file pnas.2534404123.sapp.pdf]

Supplementary Information (SI)

Materials and Methods

Cell lines

HEK-293T (ATCC, CRL-11268), VeroE6, and HeLa cells (ATCC, CCL-2) were maintained in high-glucose DMEM media supplemented with GlutaMAX® and sodium pyruvate (Cat.No.31966021), 10% fetal calf serum (FCS), and 100 U/mL penicillin/streptomycin (p/s). BSRT-7/5 CL21 cells are a clone derived from BSRT-7/5 cells sourced from K.-K. Conzelmann, Ludwig-Maximilians-Universität München, Germany. BSRT-7/5 CL21 and BHK-21 cells were maintained in GMEM (Cat.No.11710035), supplemented with 10% fetal calf serum (FCS), 10% tryptose broth (TPB), and p/s. Additionally, BSRT-7/5 CL21 cells were supplemented with 0.25 mg/mL G418. To minimize background signal, FluoroBrite DMEM (Cat.No.A1896701), supplemented with 10% FCS, was used for 3D-SIM imaging experiments. All cell lines in our experiments were cultured at 37°C with 5% CO2.

Construction of RVFV antigenomic cDNAs

Virus antigenomic cDNAs for RVFV (ZH548 strain) genomic segments (L, M and S) were synthesized with a plasmid backbone that contained constitutive elements, including a colE1 origin of replication and a kanamycin resistance marker, sequences for a T7 promoter, T7 terminator and a hepatitis delta ribozyme (GeneArt, TFS). The sequences of the antigenomic virus segments were obtained from NCBI using the following accession numbers: ZH548 L – NC_014397.1, ZH548 M- NC_014396.1, ZH548 S – NC_014395.1 15. cDNA sequences are available on request.

RVFV rescue

BSRT-7/5 CL21 cells were seeded in 6-well plates at a density of 7×10^5 cells per well on the day before transfection. Three plasmids encoding the 3 viral segment cDNAs (0.5 µg per plasmid) were transfected using Trans-IT LT-1 (Mirus Bio). Visible CPE was observed on day 3 and highly visible CPE on day 6 and the supernatant was harvested on day 6. To confirm the presence of virus and to determine viral titer a plaque assay was performed using BHK-21 cells. The recused virus was also passaged in BHK-21 cells using a multiplicity of infection (MOI) of 0.01 to produce a working stock.

Tetrazine dyes

Janelia Fluor® 549 was dissolved in DMSO to a concentration of 5 µM and aliquoted for storage at −20°C. For labeling, the stock was further diluted 1/100 to a final concentration of 50 nM. SiR-tetrazine (SiR-tz) (Spirochrome, CAT: SC005), was dissolved in DMSO to a final concentration of 0.5 mM and aliquoted for storage at −20°C. For labeling, the SiR-tz stock was diluted in PBS (for fixed cells) or cell media (for live-cell imaging) to a final concentration of 1 µM. The methyl tetrazine dye CF®500 (Biotium, CAT: 96029); was dissolved in DMSO to a concentration of 10 mg/mL and aliquoted for storage at −20°C. For labeling, this stock was diluted to 2.5 µg/mL with media before use. Tetrazine-5-TAMRA (Jena Bioscience, CLK-017-05) was dissolved in DMSO and used at a final concentration of 5 µM for labeling.

Antibodies (Ab)

A polyclonal anti-NSs Ab generated against the ZH548 NSs protein was commissioned from GenScript. A fluorescently labeled version of this Ab (Alexa Fluor® 488) was also generated by GenScript and was used for RVFV NSs WT 3D-SIM imaging. For Western blot analysis, the polyclonal anti-NSs Ab was used as the primary Ab with IRDye® 800CW goat anti-rabbit IgG secondary Ab (LI-COR, Cat.No. 926-32211). Anti-alpha-tubulin Ab (Sigma, Cat.No. T5168) was used as a loading control with IRDye® 680RD goat anti-mouse IgG secondary Ab (LI-COR, Cat.No. 926-68070).

Immunofluorescence of NSs in RVFV infected VeroE6 cells

VeroE6 cells were grown in 8-well chamber slides and infected with RVFV (ZH548) at a MOI of 0.1. After 24 h of infection, cells were fixed for 1 h with 4% formaldehyde and washed twice in PBS before being permeabilized for 3 times, 5 min each with 0.1% Triton X-100 in PBS on a shaker. The fixed cells were then blocked with 0.5% BSA in PBS for 0.5 h at room temperature on a shaker. Following blocking, cells were incubated for 1 h on a shaker at room temperature with the anti-NSs Alexa Fluor® 488 linked Ab diluted 1:500 in block solution and washed 3 times, 5 min each, in PBS before analysis by widefield imaging and 3D-SIM.

Genetic-code expansion plasmids

pcDNA3.1(+)_U6 tRNAPyl_CMV NESPylRS(AF) was a gift from Ivana Nikić-Spiegel (Addgene plasmid # 182287; http://n2t.net/addgene:182287; RRID:Addgene_182287). Constitutive over-expression vectors containing the DNA coding sequence of NSs derived from the RVFV strain ZH548 (GenBank: NC_014395.1) with amber stop codon (TAG) substitutions (S99, K108, S112, Q138, Q140, Q199, K202, S206, S226) were purchased from Twist Bioscience (pTwist CMV wPRE BG Neo NSs). A plasmid containing the WT NSs sequence was created using the QuikChange® protocol (Stratagene). Additional amber stop codon substitutions (Q12, K84, S119, S126, S127, and K150) were also introduced into the WT NSs plasmid.

Transfection for genetic-code expansion

Transfection of HEK-293T and HeLa cells in 24-well plates and 8-well chamber slides was carried out for 24 h using Lipofectamine™ 3000 (TFS, Cat.No.L3000001) according to the manufacturer's instructions with 250 ng total DNA (125 ng pcDNA3.1(+)_*U6 tRNAPyl*_CMV NESPylRS(AF) and 125 ng pTwist CMV wPRE BG Neo NSs). Typically, for wide-field imaging, HeLa cells were seeded at a density of 2×10^4 cells per well in 8-well chamber slides with a glass bottom (Ibidi, Cat.No.80807) or a tissue culture-treated polymer surface (Ibidi Cat.No.80806). For 3D-SIM, glass-bottomed 8-well chamber slides were coated with human fibronectin at 20 µg/mL (Merck, Cat.No. FC010). For Western blotting, HEK-293T and HeLa cells were seeded at 4×10^4 cells per well in 24-well plates and transfected with 500 ng total DNA (250 ng pcDNA3.1(+)_*U6 tRNAPyl*_CMV NESPylRS(AF) and 250 ng pTwist CMV wPRE BG Neo NSs). Trans-Cyclooct-2-en-L-Lysine (TCO*A; SiChem, Cat.No. SC-8008) was dissolved to generate a 100 mM stock solution (stored at −20°C) in 0.2 M NaOH and 15% v/v DMSO. Culture media was replaced with media supplemented with 250 µM TCO*A 10 min post-transfection.

Verification of TCO*A**-dependent tetrazine labeling**

To assess whether tetrazine dyes bind non-specifically to WT RVFV NSs filaments in the absence of TCO*A incorporation, HeLa cells were seeded on glass coverslips in 12-well plates at a density of 1×10^5 cells per well. Cells were transfected with 1000 ng of pTwist CMV wPRE BG Neo NSs (WT) using Lipofectamine™ 3000 (TFS, Cat.No.L3000001) according to the manufacturer's instructions for 24 h in the absence of TCO*A. Following transfection, the media was removed, and cells were washed three times in PBS before fixation for 10 min with 4% paraformaldehyde. Cells were then washed three time in PBS and permeabilized once for 5 min with 0.1% Triton X-100 in PBS. Following permeabilization, cells were blocked overnight at 4°C in media containing 10% FCS. The next day, cells were incubated for 30 min at room temperature with either 50 nM JF549 or 1 µM SiR-tz in PBS. Cells were then washed three times in PBS and incubated for a further 3 h in PBS on a shaker at room temperature. Since NSs filaments lack TCO*A incorporation and therefore cannot be labeled with tetrazine dyes, cells were subsequently incubated overnight at 4°C with anti-NSs Alexa Fluor® 488 Ab diluted 1:500 in blocking solution of 5% FCS in PBS to enable visualization of NSs filaments. The following day, cells were washed three times in PBS, mounted on glass slides with mounting medium (ProLong™ Diamond Antifade Mountant, P36965), and imaged by widefield microscopy on a Leica DMi8 microscope using a 40× dry objective (NA 0.75). Alexa Fluor® 488 fluorescence (green channel) was observed, confirming the presence of NSs filaments, while no fluorescence was detected in the yellow or far-red channels for Janelia Fluor® 549 or SiR-tz, respectively. This demonstrates that tetrazine dyes do not bind

non-specifically to wild-type NSs filaments and that TCO*A incorporation is required for tetrazine-based labeling.

Widefield fluorescence microscopy of control samples

To analyse NSs filament formation in control samples, cells stained with anti-NSs Alexa Fluor® 488 Ab and incubated with tetrazine dyes were imaged on a Leica DMi8 widefield microscope using a 63× oil-immersion objective (NA 1.25). Acquisition settings were: Alexa Fluor® 488 (FITC), 5 ms exposure, gain 5; JF549 (yellow), 1 s exposure, gain 5; SiR-tz (far-red), 1 s exposure, gain 5.

To image WT RVFV (ZH548) NSs filaments, VeroE6 cells were stained with anti-NSs Alexa Fluor® 488 Ab as described in the immunofluorescence section and imaged on a Leica DMi8 inverted widefield microscope using a 40× dry objective (NA 0.60). Images were acquired using the FITC channel (1s exposure).

Western blotting

RVFV NSs samples for polyacrylamide gel electrophoresis (PAGE) were produced in HEK-293T and HeLa cells. Cells grown in 24-well plates were lysed 24 h post-transfection using 100 µl per well of Bolt® LDS sample buffer (Thermo Fisher Scientific, Cat.No. B0007) supplemented with Bolt® reducing agent (Cat.No. B0009) before incubation at 95°C for 10 min. Samples were then stored at −20°C before use. Before PAGE, samples were incubated at 85°C for 5 min prior to loading on 4-12% NuPAGE Bis-Tris gels (Thermo Fisher Scientific, Cat.No. NW04122BOX). After approximately 1 h, PAGE was stopped and proteins were transferred to a nitrocellulose membrane using a Bio-Rad semi-dry transfer system at 15 V for 30 min. The membrane was blocked using Intercept® (PBS) Blocking Buffer (LI-COR) and proteins were visualized on a LI-COR Odyssey CLx imager according to the manufacturer's instructions. Chameleon Duo pre-stained molecular weight markers were purchased from LI-COR (Cat.No.928-60000).

Imaging RVFV NSs in fixed cells

RVFV NSs-expressing cells were transfected with pTwist CMV wPRE BG Neo NSs and pcDNA3.1(+)_*U6 tRNAPyl*_CMV NESPylRS(AF) in 8-well chamber slides as detailed above in the presence of TCO*A and incubated for 24 h. To wash out TCO*A and reduce background, cell media was removed, and cells were washed twice with pre-warmed media before incubation in fresh media for 2–3 h. Next, cells were washed twice in PBS before fixation for 20 min with 4% formaldehyde. Cells were then washed twice in PBS and permeabilized 3 times for 5 min each with 0.1% Triton X-100 in PBS on a shaker. Cells were then incubated for 30 min at 37°C with 50 nM JF549 or 5 µM Tetrazine-5-TAMRA. To wash out unbound dye, the dye solution was aspirated, and cells were rinsed three times in PBS prior to washing with PBS on a shaker at room temperature for 30 min. Fluorescence was visualized by 3D-SIM immediately, or alternatively, mounting medium (Ibidi, Cat.No. 50001) was added and chamber slides were stored at 4°C in the dark until imaging.

Live-cell 3D-SIM

HeLa cells were transfected with pTwist CMV wPRE BG Neo NSs and pcDNA3.1(+)_*U6 tRNAPyl*_CMV NESPylRS(AF) for 24 h as above in the presence of TCO*A. To wash out TCO*A, cell media was removed, and cells were washed twice with pre-warmed media before incubation in fresh media for 2–3 h at 37°C. The media was then replaced with media containing SiR-tz at 1 mM and incubated for 30 min at 37°C. The media was then replaced with media containing SiR-tz at 1 µM and incubated for 30 min at 37°C. For imaging, cells were washed twice with fresh media and incubated for a further 2–3 h at 37°C to remove unbound dye. The media was then replaced with FluoroBrite™ DMEM containing 10% FCS and incubated typically for 1–2 h before live-cell 3D-SIM imaging to further minimize background signal.

Widefield fluorescence microscopy

To analyze amber stop codon suppression by screening for NSs filament formation, chamber slides containing cells expressing RVFV NSs filaments labeled with tetrazine dyes or stained with an anti-NSs Alexa Fluor® 488 Ab were analysed on a Leica DMi8 widefield microscope using a 40× dry objective (NA 0.6).

3D-SIM

Samples in chamber slides were imaged using a Zeiss Elyra 7 lattice SIM microscope using ZEN Black 3.0 software, with either a 63× 1.4NA oil immersion or 63× 1.2NA water immersion objective. Lattices used were: for Alexa Fluor 488-stained samples G5 (grating period 27.5 μm); Janelia Fluor® 549 samples G4 (32 μm) or G5 (27.5 μm if phase modulation was judged to be sufficiently good); SiR-Tz (647 nm) G3 (36.5 μm), all using 13 phases. Z-stacks were captured at intervals of 91–125 nm depending on the excitation wavelength; or for some samples at 55 nm (over-sampling). Lasers (488 nm, 561 nm and 642 nm, all 500 mW) were typically used at 2.5–4% power. Processing was by SIM^2 [1] using "standard live" settings of input SNR medium, iterations 16, regularization 0.065, input and output sampling set to ×4, median filter. Grating period was 718.33 nm and resultant xy scaling 0.031 μm. Additional filaments labeled with Janelia Fluor® 549 were imaged on a Zeiss Lattice SIM 3 using Zen Blue software with a 40×/1.4 NA oil immersion objective. Images were captured in SIM-apotome mode, with a 26.8 μm grating and 5 phases. Z-stacks were captured at intervals of 138 nm and a 561 nm laser was used at 5–8% power. Processing was performed using SIM^2 in 3D mode with Wiener filter order recombination, a sharpness filter of 10.5746, no regularization, 8 iterations, with ×4 processing and ×4 output sampling.

AI powered filament tracing using Imaris

Processed 3D-SIM image data files were imported into the Imaris image analysis software (version 10.2.0, Oxford Instruments) by converting ZEISS CZI files to Imaris IMS files using the Imaris File Converter (version 10.2.0). To reduce background noise and improve image quality before filament tracing, deconvolution was performed in Imaris using robust deconvolution with 20 iterations. The acquisition parameters input were: TIRF for imaging modality, 1.4 for objective lens numerical aperture, 1.37 for refractive index with oil immersion medium, and 500 nm imaging distance from the coverslip.

The AI-powered Imaris Filament Tracer module was applied to the 3D-SIM data to automatically detect and segment RVFV NSs fluorescence in 3D. The algorithm settings used were AutoPath (no loops) without soma or spines for filament detection. We applied 3D cropping of the region of interest to reduce processing time and selected a minimum filament diameter of 50 nm (based on filament diameter in 3D-SIM) with no maximum diameter constraint. To define NSs fibers, seed points were added manually, and an iterative machine-learning process of adding and removing seed points to fluorescent structures was performed until the isosurface converged and further iterations did not significantly change its shape. Manual validation was performed by 2 independent observers, and all analyses were performed in duplicate. Finally, for final refinement of the isosurface, we used the fluorescence intensity thresholding tool to allow manual analysis of 3D-SIM fluorescence. Disconnected segments were removed and individual microfilaments were added manually.

Measurements of NSs polygons

We defined a "small" filament as one composed of one or two parallel rows of polygonal structures along the long axis of the filament. Filaments with more complex heterogeneous architecture were termed "large." Measurements of the longest dimension were performed using Imaris. Measurement points were represented as red spheres and positioned at the center of filament isosurface lines that corresponded to the lines of maximal fluorescence intensity. When filament lines did not coincide with fluorescence intensity lines, measurement points were instead added to the center of the fluorescence intensity lines only, using the manual selection option in the Imaris Filament Tracer program.

To quantify network architecture, we counted the number of junctions (defined as branch points where three or more filaments converge) forming polygonal holes (defined as enclosed spaces surrounded by filament segments for each labeling condition (JF549, SiR-Tz, and Alexa Fluor® 488) using filament isosurface and fluorescence intensity line overlays displayed in the Imaris software.

It is interesting to speculate that small filaments might represent early infection structures optimised for initial host targeting, while large filaments could represent mature structures with enhanced capacity for complex regulatory functions.

Table 1.

Filament type	Number of filaments imaged by 3D-SIM
Live-cell (GCE)	30
Fixed (GCE)	15
WT overexpressed (anti-NSs Alexa Fluor 488)	17
RVFV infected (anti-NSs Alexa Fluor 488)	32

SI Appendix Reference

1. A. Löschberger, et al., Super-resolution imaging by dual iterative structured illumination microscopy. *bioRxiv* [Preprint] (2021). https://doi.org/10.1101/2021.05.12.443720 (accessed 12/02/2026).
